# Techno-economic analysis of natural gas distribution using a small-scale liquefied natural gas carrier

**DOI:** 10.1038/s41598-023-50155-8

**Published:** 2023-12-21

**Authors:** Muhammad Arif Budiyanto, Gerry Liston Putra, Achmad Riadi, Gunawan, Apri Melianes Febri, Gerasimos Theotokatos

**Affiliations:** 1 Department of Mechanical Engineering, Universitas Indonesia, Kampus Baru UI Depok, Jawa Barat, Indonesia; 2 Department of Naval Architecture, Ocean and Marine Engineering, University of Strathclyde, Glasgow, UK

**Keywords:** Environmental economics, Socioeconomic scenarios, Sustainability

## Abstract

The design of the gas distribution for small-demand power plants located on remote islands is logistically challenging. The use of small-scale liquefied natural gas (LNG) vessels can be an option for these logistic problems. This paper aims to conduct a techno-economic analysis of using small-scale LNG vessels for gas distribution to the power plants that are spread across different islands. Route optimisation has been conducted using the capacitated vehicle routing problem method. The ship’s principal dimensions were determined using the aspect ratio from a linear regression of existing small-scale LNG vessels. As a case study, the gas demands for a gas power plant in eastern Indonesia were analysed into four distribution clusters. The results of the techno-economic analysis showed that the four distribution clusters have different characteristics regarding the LNG requirements, location characteristics and ship specifications. The capacity of small-scale LNG vessels feasible in terms of technical aspects varies from 2500, 5000, to > 10,000 m^3^ with variations in the ship speed depending on the location of the power plants. The amount of cargo requested and the shipping distance was affected to the cost of LNG transportation. The economic assessment proposes that the feasible investment by considering small-scale LNG cargo distribution, from the case study shows that with a ship capacity of 5000 m^3^ feasible margin rate is ≥ 3 USD/metric million British thermal units with an internal rate of return of 10% and estimated payback period is less than 15 years.

## Introduction

The global supply of liquefied natural gas (LNG) is a profitable trend with the growing use of natural gas as a cleaner fossil fuel for various industries and households. The global LNG market demand increased by 356.06 million tonnes in 2019, with a compound annual growth rate (CAGR) of 5.8% predicted from 2020 to 2027^1^. Natural gas demand has recently risen dramatically in Asia^2^. LNG is used as a transportation fuel as well as in the power and electricity generating, heating, cooling and various industrial uses^3,4^, indicating that it may be used to replace crude oil in various situations^5,6^. LNG has recently emerged as a lower carbon emissions energy source for the generation of electricity^7^. Small-scale and mobile power plants have been developed by several power plant manufacturers^8^. Between 2017 and 2022, the global mobile power generation market is expected to increase at a CAGR of 4.56%, resulting in a market value of USD 1.73 billion^9^. The increased investment in electrification and electricity in remote areas will propel the market forward^10,11^.

Small-scale LNG vessels are a special case of gas supply chains for mobile power plants^12^. Several challenges in optimising the LNG supply chain network exist, e.g. price and demand volatility^13^, different gas production possibilities^14^, varied distribution modes^15^, variable navigational paths^15^, fluctuating annual demand^16^ and optimising infrastructure construction and inventory routing^17^. Due to the intricacy of the LNG supply chain, optimising the LNG supply system will be tough; therefore, solving this problem will be difficult^18^.

Small-scale LNG routes are a unique combination of the maritime inventory route issues and a number of extra factors, including changing production and demand levels, as well as port terminal constraints^19,20^. Most of the researchers have been working on the subject of vehicle routing or vehicle routing challenges to tackle the maritime distribution issues^21^. Vehicle routing difficulties involve establishing the best route for a problem requiring multiple vehicles with a limited capacity to serve various clients based on their requirements^22,23^.

Various vehicle routing challenges and ways for solving them were noted^24^. The branch-cut-and-price approach^25^, which is commonly used to address the land transportation problems, is a type of vehicle routing problem that has been widely employed to handle the capacity-based transportation cases^26^. In this study, a capacitated vehicle routing problem, a variant of the vehicle routing problem, in which each vehicle has a limited capacity and must service requests from the depots with a single type of goods from the distributor while minimising the total travelled distance was considered^27^. Few studies have applied the problem of determining the capacity and route of the vehicles of the small-scale LNG vessels^28^. Research on small-scale LNG carriers is an important area of study due to the growing demand for LNG as a cleaner energy source and the need to efficiently transport LNG to various markets, including remote or niche locations.

There are at least two research gaps that must be addressed for LNG transportation in remote areas: design and economics. Research gaps in the design of small-scale LNG carriers include optimal use of small-scale shallow-draft LNG carriers and LNG as a fuel for LNG carriers^29,30^. Comparative economic analysis of LNG distribution to power plants taking into account elements like investment costs, operational expenses, and profit margins is one of the research gaps in small-scale LNG economic analysis^31^. To answer these two research gaps, a connected study is needed between the technical aspects of small-scale LNG ships and their economic analysis.

This study aims to determine the best route, find the optimum ship dimension and calculate an overview for an economic analysis based on the financial feasibility parameters for the LNG distribution. Moreover, this study contributes to a better understanding of the best allocation of a small-scale LNG distribution for the small-scale power plants as well as a comprehensive case study of the gas power plants for eastern Indonesia.

## Research methods

### Methodology framework of techno-economic analysis small-scale LNG carrier

**Figure 1:**
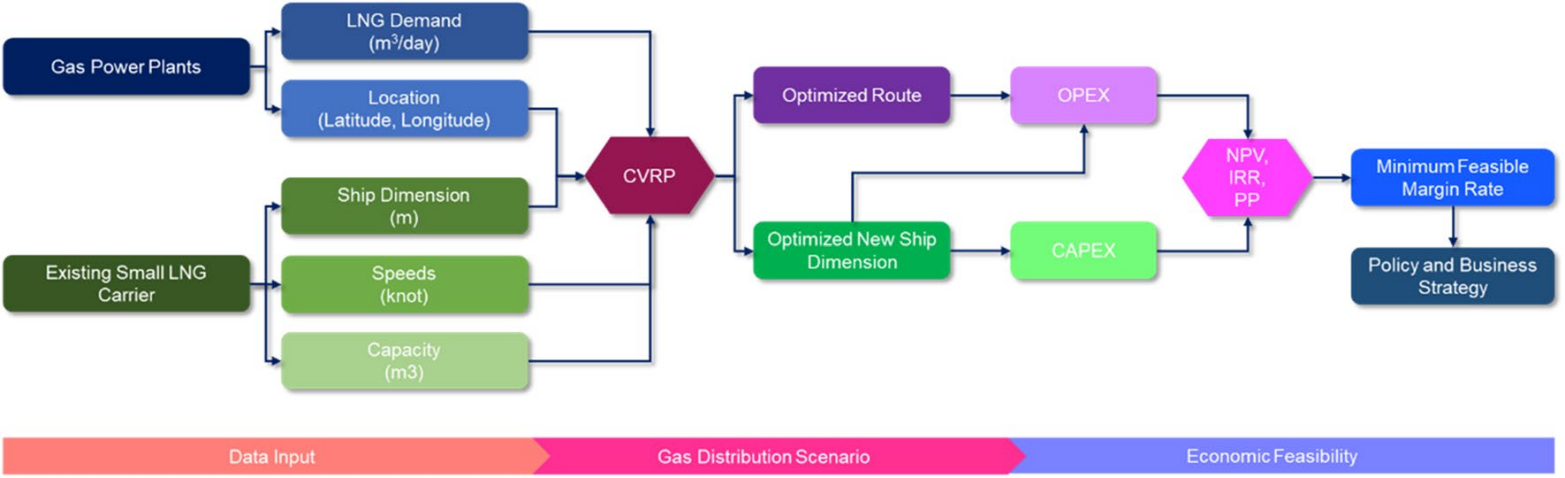
Methodology of techno-economic analysis of Small-scale LNG Carrier.

This study uses a theoretical and practical approach that starts from the energy demand of gas power plants juxtaposed with existing LNG vessels, finds the optimal route and dimensions of the new ship, and then completes the calculation of its economies of feasibility. A systematic methodology has been designed to answer whether the routes and dimensions of the new LNG vessels are feasible to implement from an economic standpoint. The systematic method in this study is shown in Fig. 1. The methodology used is categorized into three stages, namely data input, gas distribution scenario, and economic feasibility. The input data used consists of LNG demand data for gas power plants and data on existing small LNG carrier vessels. The data is then processed using the capacitated vehicle route problem which will produce the optimal route and ship size recommendations. From these results, the economic feasibility is then assessed to determine the minimum feasible margin rate and the required business and policy strategy. Each stage will be explained in detail in the next section.

### LNG demands from gas power plants

LNG demands were conducted by collecting data to obtain an overview of the existing system. Secondary data collection is obtained from the institutions that publish data on the digital systems, e.g. websites owned by the annual reports of related companies, articles, journals and the literature studies. The data collected are of power plants, gas demands, LNG ships and data on the distance between the receiving terminals. In determining the LNG delivery scenario, knowing the source of the nearest LNG refinery to minimise shipping costs is necessary. In this study, power plant data were taken from the state electricity company 2017–2026 electricity supply business plan^32^. The electricity supply business plan is a planning document prepared by the state electricity company in Indonesia that contains medium to long-term strategies and plans to meet national electricity needs, including the development of power generation, transmission, and distribution of electricity.

**Figure 2:**
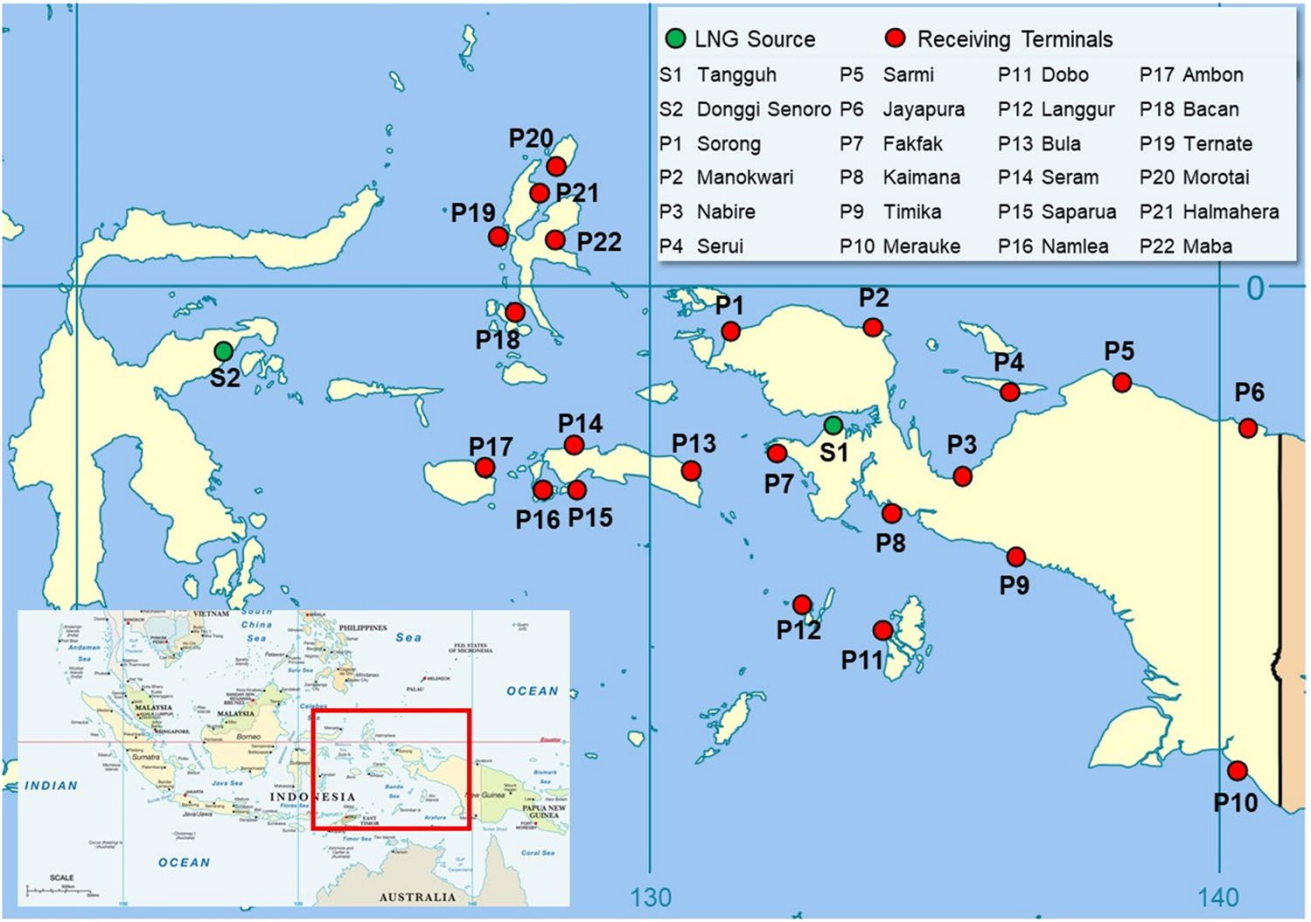
Location of LNG sources and receiving terminals.

Power plant data used in this study is of the power plants using small-capacity gas fuels located in eastern Indonesia. Two main LNG sources were used in this study, i.e. the Tangguh LNG plant in Bintuni Bay, Papua and Donggi Senoro LNG in Matui, Sulawesi. The selection of LNG sources is based on the geographical location close to the power plant clusters in eastern Indonesia. In addition, the production capacity of the LNG plant is sufficient for the demand for power plants in the area. The identification of the receiving terminal is also conducted by determining the location of the receiving terminal with the help of an electronic map. From this identification, a distance matrix between the receiving terminals can then be made. Figure 2 shows an electronic map of the distribution data of the LNG sources and the receiving terminals. Figure 2 was created by combining LNG receiving terminal location data with modified maps from Wikimedia Commons^33^ and VectorStock^34^.

LNG demand is done by calculating the fuel consumption of each power plant per day. However, each adjacent power plant located in one area is made into one terminal as a representative of each region to classify the LNG demand data. Thus, the data is represented as LNG consumption per day in each region are shown in Table 1.

**Table 1:** LNG demands of gas power plant in eastern region of Indonesia^32^.

	Location	BBTU	MMSCFD	LNG demand (m^3^/day)
P1	Sorong	12	10.5	549.6
P2	Manokwari	7.2	6.3	329.8
P3	Nabire	6	5.2	274.8
P4	Serui	2.6	2.3	119.1
P5	Sarmi	0.6	0.5	27.5
P6	Jayapura	13.2	11.5	604.6
P7	Fakfak	2.4	2.1	109.9
P8	Kaimana	1.2	1	55
P9	Timika	2.6	2.3	119.1
P10	Merauke	4.8	4.2	219.9
P11	Dobo	0.6	0.5	27.5
P12	Langgur	2.4	2.1	109.9
P13	Bula	1.2	1	55
P14	Seram	4.5	3.9	206.1
P15	Saparua	1.2	1	55
P16	Namlea	0.6	0.5	27.5
P17	Ambon	2.1	1.8	96.2
P18	Bacan	2.4	2.1	109.9
P19	Ternate	8.4	7.3	384.7
P20	Morotai	1.2	1	55
P21	Halmahera	4.1	3.6	187.8
P22	Maba	1.2	1	55

### Indentification of existing small LNG carrier

At this stage, the current study uses the assumption of ship capacity as a limitation in the optimisation calculations. This can be an illustration of the maximum capacity that can be transported. Determining the best ship dimensions can be done in addition to obtaining the best capacity on the ship. To identify the carrier vessels, the researchers collected some comparative ship data sourced from various ship registration websites from the world classification bodies. The dimensions of the ship are then recorded to be used as calculations in determining the dimensions of the new ship based on the planned capacity. A statistical method is used to solve single problems, which is very suitable for engineering projects for adjusting modern ship data based on the analytical research^35^. In general, the analytical data based on the principal dimensions of a ship which consist of length (L), beam (B), draught (T) and height (H) as well the coefficients of a ship's form i.e., block coefficient (Cb), midship section coefficient (Cm), waterplane coefficient (Cw), and prismatic coefficient (Cp). The data collected can be aspect ratios L/B, B/T and L/T as criteria parameters on similar ships or ships of similar model and type but different sizes^36^.

The data collected from the experimental results of various types of ships are depicted on an L/B ratio curve of course with other factors that are closely related to the aspect ratio, e.g. Cb, Cw, Cm and Cp, which all provide a concrete estimate of the speed, power and carrying capacity of the model ship. The comparison ship data collection was taken from several websites of the world classification bodies, which have data on small-scale gas carriers. Some of these classification bodies include Nippon Kaiji Kyokai^37^, Det Norske Veritas^38^, Bureu Veritas^39^, Korean register^40^, Lloyd's Register^41^ and the Indonesian Classification Bureau^42^. Data for small-scale gas carriers that have been obtained from the classification agency are shown in Table 2.

**Table 2:** Particular dimension of small-scale LNG carrier.

	Ship names	Principal dimension (m)				Speeds (kn)	Capacity (CBM)	Data references
		Length (L)	Beam (B)	Height (H)	Draught (T)			
1	Pioneer Knutsen	63.4	11.83	5.5	3.5	12	1100	Nippon Kaiji Kyokai^37^, Det Norske Veritas^38^, Bureu Veritas^39^, Korean register^40^, Lloyd's Register^41^ and the Indonesian Classification Bureau^42^
2	K Gas	64	12.8	5.5	4.7	14	1838	
3	North Pioneer	83	15.3	7.2	4.312	14.8	2512	
4	Kakurei Maru	80.3	15.1	7	4.312	14.6	2536	
5	Shinju Maru No.2	80.3	15.1	7	4.198	14.5	2536	
6	Shinju Maru No.1	80.3	15.1	7	4.183	14.6	2538	
7	Kakuyu Maru	82.56	15.3	7.2	4.313	14.9	2539	
8	WSD59 3 K	82.6	15.2	8	4.6	12	3000	
9	No.1 SJ Gas	89	14.8	7	5.7	13.9	3017	
10	Miracle Road	90.1	15	7.2	4.8	15.2	3266	
11	Jisan Gas	92.5	15.8	7.1	5.5	12.3	3313	
12	Asia Gas	87.66	17.6	7.1	4.2	13	3516	
13	Shining Road	90.09	16.5	7.2	5.4	14.5	3535	
14	Gas Lotus	89.9	16.5	7.2	5.413	13.2	3535	
15	Lady Rosalia	89.24	16.5	7.2	5.533	13	3544	
16	Akebono Maru	93	17.2	7.8	4.613	15.8	3587	
17	LNG Oil Combi	95.04	15.4	9.6	5.4	14	4517	
18	WSD59 5 k	94.5	18.4	9.3	5.7	14	5000	
19	Schumi 5	99.97	19.6	7.7	5.514	12	5001	
20	DL Lily	100.24	17.6	8.1	5.784	13.5	5018	
21	JS Cougar	92.5	17.4	11.7	7.2	15	5034	
22	WSD59 6.5 k	94.5	19.2	9.62	5.8	13	6500	
23	Coral Methane	108	18.6	9.7	6.8	15.5	7500	
24	Wsd50 7.5 k	109	18.6	10.85	5.8	15	7500	
25	Gas Ionian	112.58	19.8	11.2	5.6	15.2	9120	
26	WSD59 10 K	117.2	22.4	11.8	6.6	14	10,000	
27	WSD55 12 K	129.3	22.4	11.8	6.2	14.5	12,000	
28	Pelita Energy	125	25.7	13.1	7.116	16	18,942	
29	Wsd50 20 k	138.7	25.3	17.6	7.8	15	20,000	
30	Triputra	145.17	28	12.57	7.06	17	23,097	

### Capacitated vehicle routing problem

The LNG distribution challenge is classified as a capacitated vehicle route problem (CVRP). CVRP refers to a vehicle routing challenge in which vehicles with limited carrying capacity must pick up or deliver things at many locations^43^. LNG carrier capacity will be a constraint for LNG transportation^44^. Route results and shipping costs will vary because each LNG ship has a different capacity employed. Given the distance between site *i* and location *j* (*S*_*ij*_), demand for each receiving terminal (*D*_*i*_), LNG ship’s loading capacity (*Q*) and the transportation costs (*C*_*ijk*_) for all routes (boat charter, fuel and port fee) are all calculated using the CVRP model. *X*_*ijk*_ represents the decision variable in this study, where ship *k* will transfer LNG along the route (R) from location *i* to location *j*, with *i*, *j* = {0,1, 2,…,* |*R*|*}, *i*, *j* ∈ *R*, *i* ≠ *j* and *k* = {1,2,…,*|K|*}, *k* ∈ *K*. Location 0 represents the LNG source. If ship *k* transports LNG from location *i* to location *j*, then the value of *X*_*ijk*_ becomes 1; otherwise, its value is 0.

1 $${X}_{ijk}=\left\{\begin{array}{c}1, if the ship k transport LNG from i to location j\\ 0, otherwise\end{array}\right.$$

The objective function in this study is to minimize the LNG transportation cost. It is equivalent with maximizing the utilized LNG vessel capacity with the cargo to reduce the number of used ships for the LNG transportation. The objective function is presented in Eq. (2).

2 $$min{\sum }_{k\in {\text{K}}}{\sum }_{i\in {\text{R}}}{\sum }_{j\in {\text{R}},{\text{j}}\ne {\text{i}}}{X}_{ijk}{S}_{ij}{C}_{ijk}$$

With the constraints as follows:

3 $${\sum }_{i\in {\text{R}}}{D}_{i}{\sum }_{j\in {\text{R}},{\text{j}}\ne {\text{i}}}{X}_{ijk}\le Q,{\forall }_{k}=\{1,\dots ,\left|K\right|\}$$

The constraint in Eq. (3) is the fulfillment of demand for each destination of the receiving terminal. Equation (3) also ensure that the total demands of the visited terminal served by each ship must be less than or equal to the ship’s loading capacity.

4 $${\sum }_{k\in {\text{K}}}{\sum }_{i\in {\text{R}},{\text{j}}\ne {\text{i}}}{X}_{ijk}=1,{\forall }_{i}=\{\mathrm{0,1},\dots ,\left|R\right|\}$$

Equation (4) ensure that each receiving terminal is serviced exactly once by one ship.

5 $${\sum }_{j\in {\text{R}}}{X}_{0jk}=1,{\forall }_{k}=\{1,\dots ,\left|K\right|\}$$

6 $${\sum }_{i\in {\text{R}}}{X}_{i0k}=1,{\forall }_{k}=\{1,\dots ,\left|K\right|\}$$

Equations (5) and (6) ensure that each ship route starts from the LNG source and ends back at the LNG source after transporting the LNG.

7 $$\mathop \sum \limits_{{i \in {\text{R}},{\text{i}} \ne {\text{h}}}} X_{ihk} - \mathop \sum \limits_{{j \in {\text{R}},{\text{j}} \ne {\text{h}}}} X_{hjk} = 0,\,\,\,\forall_{h} = \left\{ {1, \ldots ,\left| R \right|} \right\},\forall_{k} = \left\{ {1, \ldots ,\left| K \right|} \right\}$$

Equation (7) ensure the continuation of the route for the LNG distribution. It means that every ship that has finished servicing a receiving terminal must leave the terminal to continue distributing LNG or return to the LNG source. It is also called as the flow conservation constraint.

8 $${X}_{ijk}\in \left\{\mathrm{0,1}\right\},{\forall }_{i,j}=\left\{\mathrm{0,1},2,\dots ,\left|R\right|\right\},i,j\in {\text{R}},{\text{i}}\ne {\text{j}},\forall k=\left\{1,\dots ,\left|K\right|\right\},k\in K$$

The limit in Eq. (8) is to ensure that the decision variable used only uses an integer, 0 or 1.

### Route optimization of LNG distribution

Route optimization in this study uses the greedy algorithm to generate the possibility of all LNG distribution routes that may be formed, then one of the routes with supply needs close to the capacity that can be transported by the Small-Scale LNG Carrier will be selected. The greedy algorithm is a heuristic algorithm, which is used to solve optimization problems. The optimum solution is the solution that has the minimum or maximum value from a set of possible alternative solutions. The approach used in the greedy algorithm is to make a choice that seems to give the best return, namely by making a local optimum choice at each step with the hope that along with the optimization process, it will lead to a global optimum solution.

The LNG demand data for the gas power plant in the previous section is the input data for optimizing distribution. To design an optimization model for the LNG distribution route in this study there are several main components, namely LNG source, LNG receiving terminal, LNG transport ship, distance between terminals. The LNG source and LNG receiving terminal use the LNG demand data described in section "LNG Demands from gas power plants", while the transport ship data uses the ship identification results in section "Indentification of existing small LNG carrier". Table 3 shows the distance matrix of the designed distribution cluster. The distance between LNG distribution locations is measured based on the distance matrix of sea shipping lanes. The distance matrix between the terminal and the LNG sources is counted in every location of the cluster. The unit for this distance is in nautical miles. Distance calculations can be done with the sea distance calculator^45^.

**Table 3:** Distance matrix of the designed distribution cluster.

Location	Distance matrix (nm)							
Cluster 1		S1	X1	X2	X3	X4	X5	X6
Tangguh	S1	0	116	278	280	335	466	801
Fakfak	X1	116	0	141	164	219	329	685
Kaimana	X2	278	141	0	124	139	188	579
Langgur	X3	280	164	124	0	114	297	580
Dobo	X4	335	219	139	114	0	183	466
Timika	X5	466	329	188	297	183	0	391
Merauke	X6	801	685	579	580	466	391	0

Location	Distance matrix (nm)							
Cluster 2		S1	Y1	Y2	Y3	Y4	Y5	Y6
Tangguh	S1	0	212	400	570	548	720	819
Sorong	Y1	212	0	188	358	336	508	607
Manokwari	Y2	400	188	0	170	148	320	419
Nabire	Y3	570	358	170	0	100	301	400
Serui	Y4	548	336	148	100	0	201	300
Sarmi	Y5	720	508	320	301	201	0	99
Jayapura	Y6	819	629	419	400	300	99	0

Location	Distance matrix (nm)							
Cluster 3		S1	Z1	Z2	Z3	Z4	Z5	
Tangguh	S1	0	168	217	374	365	321	
Bula	Z1	168	0	56	206	355	208	
Seram	Z2	217	56	0	150	299	343	
Namlea	Z3	374	206	150	0	149	193	
Ambon	Z4	365	355	299	149	0	44	
Separua	Z5	321	208	343	193	44	0	

Location	Distance matrix (nm)							
Cluster 4		S2	W1	W2	W3	W4	W5	
Donggi Senoro	S2	0	299	378	481	573	715	
Bacan	W1	299	0	79	182	274	416	
Ternate	W2	378	79	0	103	195	337	
Morotai	W3	481	182	103	0	92	234	
Helmahera	W4	573	274	195	92	0	142	
Maba	W5	715	416	337	234	142	0	

**Figure 3:**
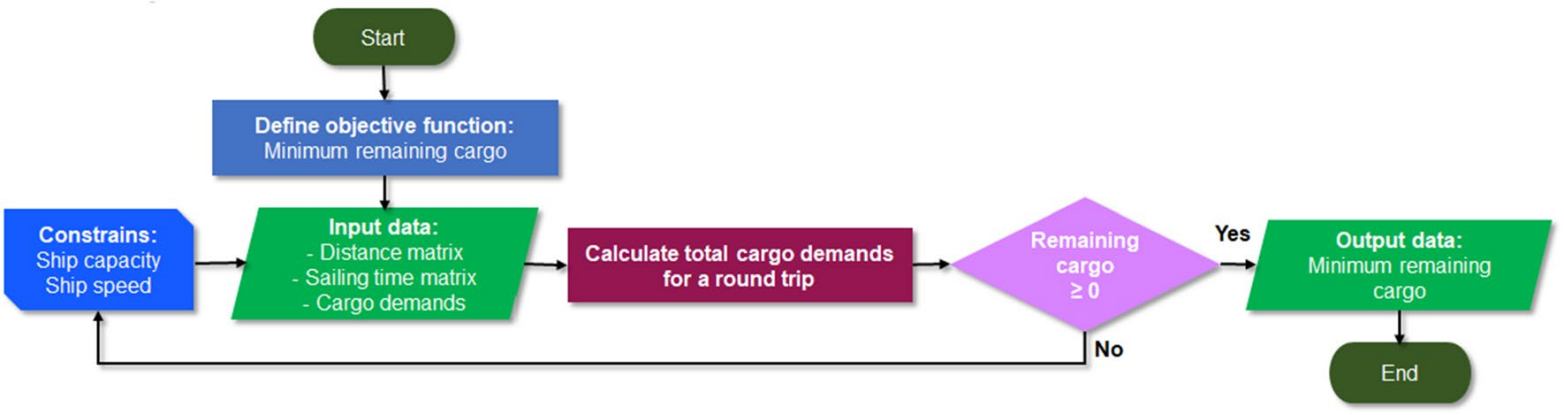
LNG distribution optimization flow.

The route optimization logic flow in this study is shown in Fig. 3. The route optimization used is a greedy algorithm with the objective function being to find the least remaining cargo value so that the ship's cargo carrying capacity is more optimal. The value in the distance matrix can be used to calculate the entire distance travelled on the possible routes that occur. The number of requests from the possible destinations that occur on the selected route is then calculated. To determine the amount of capacity loaded by the ship in one roundtrip, the total destination request is multiplied by the total roundtrip (day) of the ship once sailing. This roundtrip is influenced by several derivative variables, e.g. the ship speed, distance between the receiving terminals, anchoring time and port time.

Several components, i.e. LNG depot or source, LNG receiving terminal facilities, the number of LNG requests, ship capacity and distance between terminals, need to be considered in designing the LNG distribution scenario. The distance between each distribution location is included in the optimisation distance matrix in this study, which solves the capacitated vehicle routing problem, using the clustering method for dividing the data into several clusters, and a greedy algorithm to optimise the route in each cluster. The best route generation considers the capacity of the ship for transporting the goods.

### New ship dimension optimization using parametric design

The development of the ship's parametric design resulted from the production of several ships of the same kind but sharing a single characteristic. The designer must employ parametric design methodologies to develop ships based on historical data, which will then be enhanced and evolved, due to the commonality of these criteria. This approach makes it simpler to change the form of the hull since, when one parameter is modified, the other is parameterized and might then be automatically actualized. There are three prime parameters obtained by determining the first estimate of the ship size to be create, namely volume, mass, and particular dimensions.

One of the parametric design methods is the overall sizing strategy. This method used in determining the initial size (preliminary sizing) will vary depending on the type of ship chosen. In this method, each design must be able to strike a balance between the ability of the ship to carry the weight and the volume of the load. In this case there are 2 that must be chosen namely between weigth limited or volume limited. If the selected weight is limited, the main size can be controlled by Eq. (9).

9 $$\Delta =\gamma .L.B. T.{C}_{b}.(1+s)$$

where Δ is displacement, γ is the density of water, L the length of the ship, B the width of the ship, T is water-filled ship, *CB* is the beam coefficient, and s is the shell appendage allowance. This weight limited model parameter can be used to estimate the total weight component of the ship. Determination Δ can also use Eq. (10).

10 $${C}_{DWT}=\frac{DWT}{\Delta }$$

where DWT is deadweight tonnage and *CDWT* is the coefficient of deadweight tonnage. If volume is limited, the main measure is controlled by Eq. (11).

11 $${\nabla }_{U}=\left\{L.B.H.{C}_{Bh.}.\left(1-\sigma \right)\right\}-{\nabla }_{LS}-{\nabla }_{T}$$

where ∇*U* is the load volume, H height of the ship, beam coefficient at full depth, σ structure allowance, ∇*LS* the volume of the ship with machining equipment and other light ship weight components, and ∇*T* the volume of the hull with fuel, ballast, water, and another tank.

From the equation above the aspect ratio can be find from the data on existing small LNG vessels. The aspect ratio is important because this ratio can change the main dimensions of the ship, where each ship has special features on the shape of the hull to distinguish one from the other depending on the function and purpose of the ship being built. Aspect ratios in the determination of new vessels include L/Δ, L/B, L/T and B/T.

### Economic analysis of LNG distribution

The economic analysis in this study was calculated based on the results of optimizing the LNG distribution. Economic analysis is carried out based on several parameters as financial feasibility criteria. This analysis is carried out by considering capital expenditure (CAPEX) and operational expenditure (OPEX). Thus, the investment feasibility criteria parameters in the form of Net Present Value (NPV), Payback Period (PP), and Internal Rate of Return (IRR) are calculated to analyse the financial feasibility of the overall LNG distribution.

CAPEX is the initial investment cost for build a new ship of LNG carrier. In this study the calculation of the construction of a new ship is carried out using the empirical equation shown in Eq. (12)^46^.

12 $${C}_{LNG}=-253.012+2.6{\text{PPI}}+1.8053DWT-0.01009 {DWT}^{2}+0.0000189{DWT}^{3}$$

In Eq. (10), two basic parameters are used, namely the Producer Price Index (PPI) and Deadweight Tonnage (DWT). This equation also applies to estimating the cost of building new ships for container ships, bulk carriers, tankers and LPG tankers. In this case study, new ship construction is used by considering a longer lifespan. The typical lifespan of an LNG (liquefied natural gas) tanker is around 30 to 35 years^47^. In Indonesia as Minister of Trade Regulation, the allowable age for ships operation was a maximum of 20–25 years^48^. However, this study used the lifespan assumption is 20 years due to changing market conditions and technological advancements^49,50^.

OPEX is all costs incurred to carry out operations in a certain period, in this ship operation context used in a period of one year. The operational costs incurred are in the form of local port agent expenses, crew's costs, ship repair costs, freshwater, ship docking costs, survey and maintenance costs, and overhead costs. Then the calculation of ship operational costs is added up with the cost of fuel and lubricants. Table 4 shows the operational costs associated with LNG ship transportation.

**Table 4:** Details of operational expenditures.

Item	Cost (USD)	References
Ship’s crew	426,000.00	Estimated based on Marine Labor Convention^51^
Fresh water	1560.00	Estimated based on fresh water cost on local port data 5 USD/ton^52^
Port local agent expenses	480,000.00	Assumption based on local port data^53^
Ship repair costs	4000.00	Assumption based on literature study^54^
Ship docking cost	188,000.00	Assumption based on literature study^54^
Survey and maintenance costs	40,000.00	Assumption based on literature study^54^
Overhead cost	40,000.00	Assumption based on literature study^54^
Fuel and lubricating oil	Based on the route optimization	Calculated based on fuel cost 26 USD/km ≈ 48.2 USD/NM^55^

NPV is the difference between the present value of cash inflows and the present value of cash outflows over a period. NPV is used in capital budgeting and investment planning to analyse the projected profitability of an investment or project. NPV is the result of a calculation used to find the present value of a stream of future payments. The method of calculating the NPV value uses Eq. (13). An investment is considered profitable for the company if the NPV value is more than zero, otherwise an investment is considered detrimental if the NPV value is less than zero.

13 $$NPV=\sum_{t=1}^{n}\frac{{R}_{t}}{{(1+i)}^{t}}$$

where Rt = Net cash inflows and outflows during one period t; i = Discount rate or return that can be obtained in alternative investments; t = Number of timer periods; n = total number period.

Internal Rate of Return (IRR) is an indicator of the level of efficiency of an investment. By using the IRR, the amount of discount rate that makes the NPV equal to 0 (zero). Discount rate or interest rate is used to calculate the present value of an amount of money that will be received or paid in the future. This discount rate is used in financial analysis, investment, and asset valuation. This concept originates from the idea that the value of money in the future is less than the value of money in the present, due to various reasons such as inflation, risk, or alternative investment opportunities. If the IRR is greater than the interest rate required by the investor, then the investment is accepted. IRR in this study is calculated using Eq. (14):

14 $${\text{IRR}}={i}_{1}+ \frac{{NPV}_{1}}{{NPV}_{1}-{NPV}_{2}}({i}_{1}-{i}_{2})$$

where *i*_*1*_ = the discount rate that produces a positive NPV; *i*_*2*_ = the discount rate that produces a negative NPV; *NPV*_*1*_ = Positive NPV; *NPV*_*2*_ = Negative NPV.

Payback Period (PP) is the time required to return investment costs. PP is calculated using the ratio of initial investment costs to cash flow per year. PP in this study is calculated using Eq. (15):

15 $${\text{PP}}=\frac{Invesment cost}{Average annual cash flow}$$

In Eq. (15) the investment cost only uses the CAPEX value for building new ships. Meanwhile, the average annual cash flow uses gross income from a business activity that is carried out. In the LNG distribution business, revenue comes from the profit from the difference between the purchase price and the selling price of LNG or what is known as the sales margin rate. This study uses three variations of margin rates, namely from US$ 2 to US$ 6 per MMBtu margin. The sales margin is then multiplied by the amount of LNG sold in one year to obtain revenue per sales margin. To find out whether the investment that has been spent obtains optimum profit to determine the selling price of LNG is not too high but the LNG distribution company still earns a profit.

## Result and discussion

### Result of LNG route optimisation

**Figure 4:**
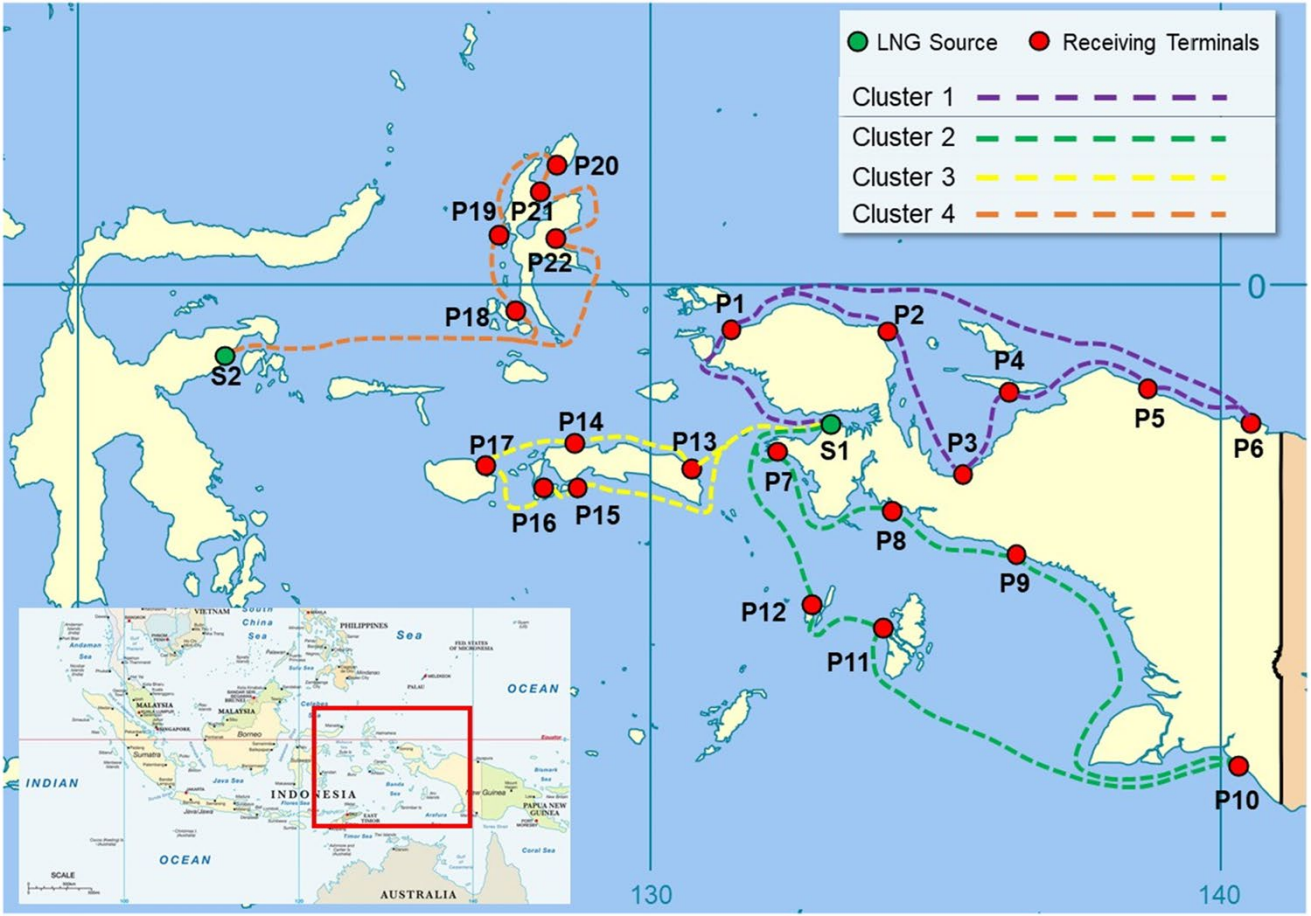
LNG clustering distribution.

In this study, the data on the location of the power plant are grouped into four clusters where the determination of this cluster is qualitatively shown in Fig. 4. Figure 4 was created by combining LNG distribution route with modified maps from Wikimedia Commons^33^ and VectorStock^34^. The determination is based on the consideration of the distance between the supplier and the generator and the geographical location of the adjacent generator. The purpose of clustering is to group data with similar characteristics into a similar area and data with different characteristics into other areas. The optimisation of the LNG distribution route is influenced by the type and the distribution route of the ship. This type of ship has a load capacity parameter that limits the number of receiving terminals to be served. The larger the loading capacity of the ship used; the more receiving terminals can be served in one delivery. This will also affect the shipping route of the LNG ship. In optimising the LNG distribution route, ship size variations were conducted provides different LNG distribution routes.

The optimisation of the LNG distribution route uses a greedy algorithm that considers the variable capacity, ship speed, sailing speed, distance between the terminal facilities and generator demand. The solution to the LNG distribution problem can be obtained by analysing the shortest route with the maximum loading capacity. In the current study, the following assumptions are used: the loading and unloading time of the ships is assumed to be constant at 3 h for all ships, port time is assumed to be constant at 6 h for all ships and each voyage from one location to another is added to an allocation of 3 h to overcome the uncertainty of shipping conditions^56^. From the calculated load volume, a boil of gas (BOG) value that comes from the LNG itself was noted. To determine the BOG value, the total load volume is multiplied by 0.15% and the sailing time^57,58^. BOG is a natural byproduct of the evaporation of LNG due to various factors such as heat input, tank equilibrium changes, and atmospheric pressure changes. he use of forced BOG has also been considered as a future fuel for LNG carriers, especially in light of global limitations on sulphur emissions and the need for alternative fuels^59^.

**Figure 5:**
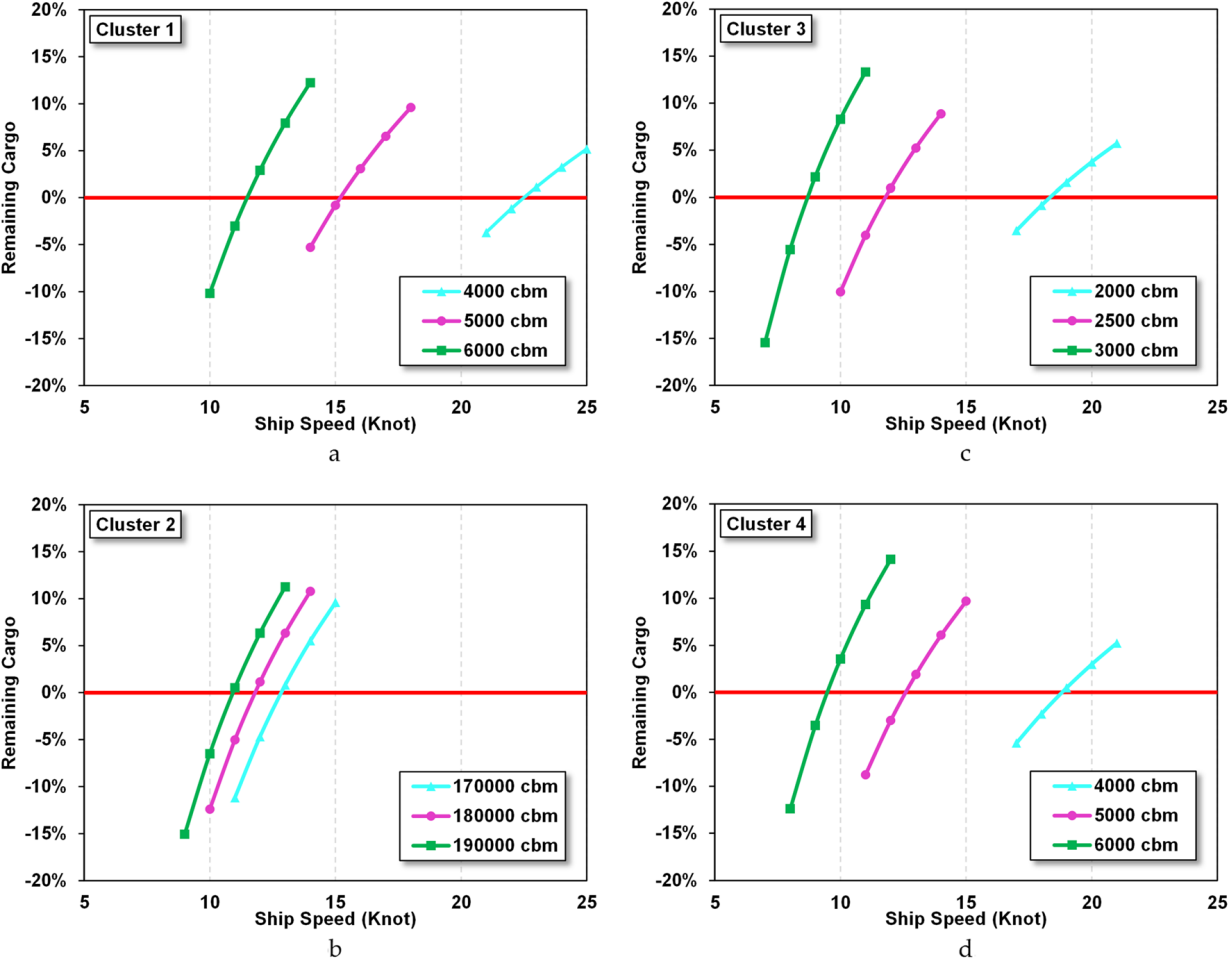
(**a**) Variation of speed vs residual charge in cluster 1. (**b**) Variation of speed vs residual charge in cluster 2. (**c**) Variation of speed vs residual charge in cluster 3. (**d**) Variation of speed vs residual charge in cluster 4.

The calculation starts by determining the selected route and then the number of requests to the selected terminal. The cargo volume is calculated from the length of sailing time of the trip on day 1 multiplied by the number of requests for each receiving terminal by considering 0.15% BOG of the original volume. The volume load is then compared with the planned ship capacity. The least residual load difference is the best solution considering the route. Figure 5a–d shows the relationship between the ship speed and remaining cargo for each power plant location using the three scenarios of small LNG vessel capacity. From this relationship, the best ship capacity and speed for clusters 1, 2, 3 and 4 were determined to be 5000, 18,000, 2500 and 5000 m^3^ with a speed of 16, 12, 12 and 13 knots, respectively. The route is determined based on the optimal capacity and speed of various route combinations. The number of shortest route combinations for each cluster varies depending on the number of destinations that have been determined. For example, for cluster 1, at least 200 combinations were carried out, and the shortest route was selected, which met the criteria that all destinations were visited by ships and there was still minimal or even close to zero remaining cargo. On the other hand, the considerations taken in determining the shortest route are also based on the average speed of the LNG carrier ship in the range of 15 knots. Even though the new LNG ship is capable of speeds of up to 21 knots in open water conditions, this has operational risks that are susceptible to being influenced by weather changes. In addition, the speed determination was chosen at medium speed because of investment costs for a new engine and its relationship with fuel consumption.

From the route optimisation, the selected route and the best ship capacity are shown in Table 5.

**Table 5:** The route selection for the LNG cluster distribution.

Cluster	Demand (m^3^/day)	Selected route	Capacity (CBM)	Speeds (knots)
Cluster 1	641.3	Tangguh → Fakfak → Kaimana → Timika → Merauke → Dobo → Langgur → Tangguh	5000	16
Cluster 2	1905.4	Tangguh → Sorong → Manokwari → Nabire → Serui → Sarmi → Jayapura → Tangguh	18,000	12
Cluster 3	439.8	Tangguh → Bula → Seram → Namlea → Ambon → Saparua → Tangguh	2500	12
Cluster 4	792.4	Donggi Sendoro → Bacan → Ternate → Morotai → Helmahera → Maba → Donggi Sendoro	5000	13

### Principal dimension of mini-LNG ships

**Figure 6:**
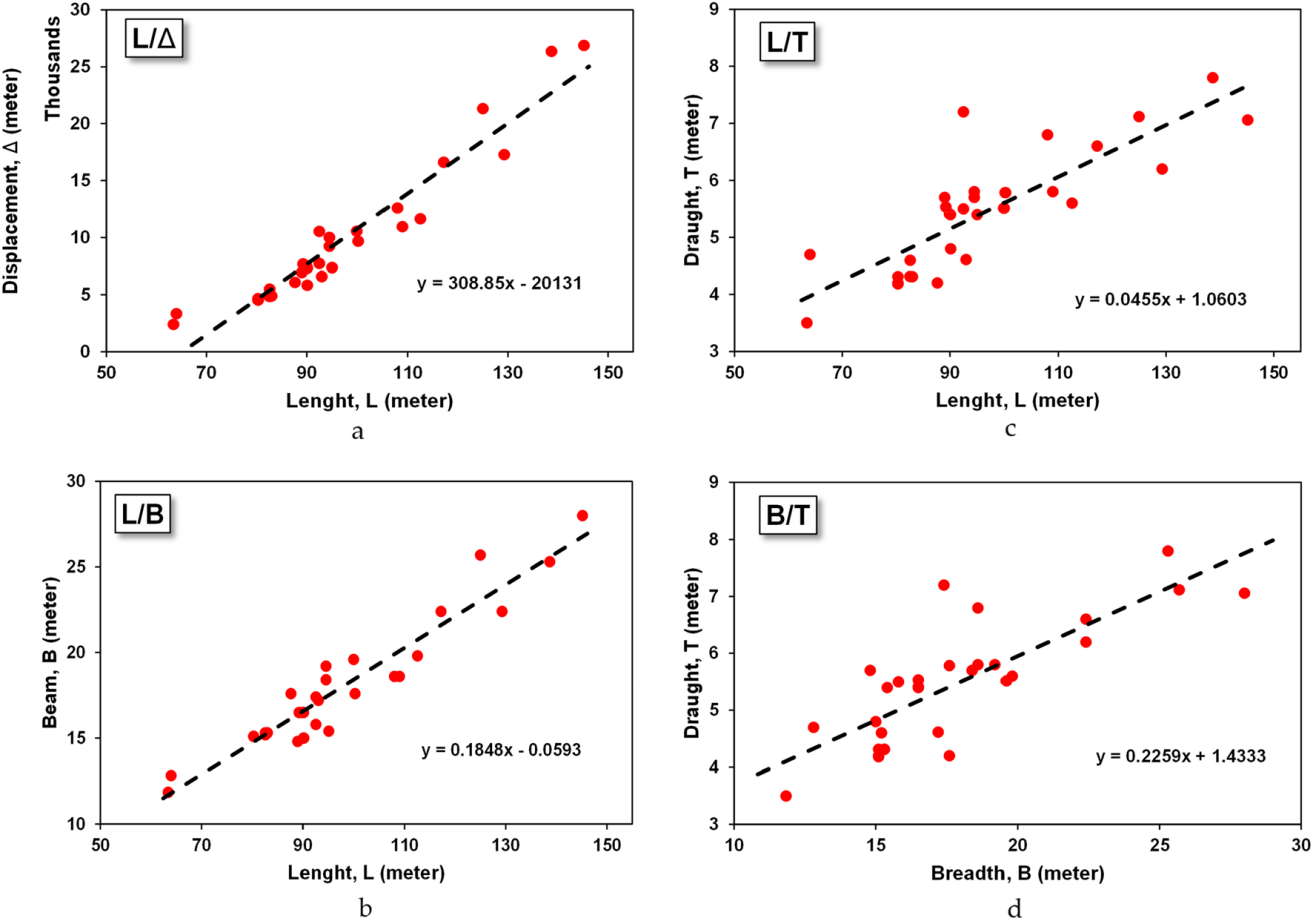
(**a**) Aspect ratio of the length-displacement of small-scale LNG vessel. (**b**) Aspect ratio of the length-beam of small-scale LNG vessel. (**c**) Aspect ratio of the length-draught of small-scale LNG vessel. (**d**) Aspect ratio of the beam-draught of small-scale LNG vessel.

Statistical methods determine the size of the ship based on the demand for available capacity. This method begins by collecting several small-scale LNG carrier vessels that have operated on at least 15 comparison vessels. From these data, a comparison is made between the dimensions of the ship and the load capacity or dimensions with the planned deadweight tonnage. Figure 6a–d are an aspect ratio between the dimensions and the capacity of the ship.

From the comparison chart of ship sizes, a linear equation can be drawn between the main dimensions and the capacity of the ship with the condition that *R*^2^ > 0.4. The capacity value can then be entered into the linear equation that has been formed earlier to produce the main dimensions of the ship. Ships whose dimensions are known are then matched with the aspect ratio of the main dimensions of the ship to find out that the dimensions of the ship have met the criteria shown in Table 6.

**Table 6:** Aspect ratio from the existing small-scale LNG ship database.

Aspect ratio	Range
L/B	4.86–6.17
L/H	7.88–13.03
L/T	12.84–20.87
B/H	1.43–2.54
B/T	2.41–4.10
T/H	0.44–0.85
T/B	0.23–0.41

The capacity value can then be entered into the linear equation that has been formed earlier to produce the main dimensions of the ship. The ship whose dimensions are known is then matched with the aspect ratio of the main dimensions of the ship to find out if the dimensions of the ship have met the criteria shown in Table 7. The data is still in the form of the ship's initial design size. The data is matched with the criteria parameters from the aspect ratio of the ship dimensions. The size of the ship that has been calculated based on this statistical method can still be optimised in terms of technicality and design economics^60^. However, for this study, it is only sufficient to estimate the initial design as a ship owner requirement.

**Table 7:** The dimension of small-scale LNG carrier for each cluster.

Ship parameters	Ship dimension			
	Cluster 1	Cluster 2	Cluster 3	Cluster 4
Capacity (cbm)	5000	18,000	2500	5000
Speed, V (knot)	16	12	12	13
Length, L (m)	92.7	134.3	84.7	92.7
Breadth, B (m)	16.8	24.6	15.3	16.8
Draught, T (m)	5.2	7.0	4.9	5.2
Height, H (m)	8.3	13.1	7.4	8.3
Block coefficient, Cb	0.9	1.0	0.9	0.9
Froude number, Fn	0.3	0.2	0.2	0.2

The dimensions of small-scale LNG carrier vessels for each cluster show that all proposed dimensions have a draft of under 10 m, even for cluster 3 the proposed vessels have a draft of under 5 m. This is very important for ship's operational aspect since in several remote area destinations there are shallow draft conditions. Delivering LNG to remote areas with limited depth of water levels in Indonesia can be challenging, with the proposed dimension small-scale LNG carriers with shallow draughts 8 m can be used to address this challenge.

### Techno-economic analysis of LNG distribution

The techno-economic analysis in this study consists of NPV, IRR and payback period by considering capital and operational expenditure where the calculation method has been explained in section "Economic analysis of LNG distribution". Table 8 shows the capital expenditure and OPEX in 1 year operation per cluster distribution. From these results, it can be seen that the largest capital costs are for cluster 2, where in this cluster the largest ship size is required with capacity of 18,000 cubic meters. Otherwise, the largest operational costs are needed in Cluster 1 because it has a long shipping route and more roundtrips than other clusters.

**Table 8:** Capital and operational expenditure of Small-scale LNG carrier.

	Newbuilding cost	Operational cost
Cluster 1	USD 177,879,685.39	USD 4,955,897.45
Cluster 2	USD 189,098,091.54	USD 4,326,947.70
Cluster 3	USD 175,620,019.79	USD 3,783,030.78
Cluster 4	USD 177,879,685.39	USD 4,397,376.45

Table 9 shows the estimated income from LNG transportation costs with several variations in margin rates over a period of 1 year of operation. Cluster 2 has the highest income in general since it has more capacity than the other clusters.

**Table 9:** Income estimation with margin rate variation.

Margin rate (USD/MMBTU)	Cluster 1	Cluster 2	Cluster 3	Cluster 4
2	–	USD 30,359,472.15	–	–
3	USD 15,982,510.58	USD 45,539,208.23	–	USD 23,535,888.87
4	USD 21,310,014.11	USD 60,718,944.30	USD 14,012,064.07	USD 31,381,185.16
5	USD 26,637,517.63	–	USD 17,515,080.09	USD 39,226,481.45
6	–	–	USD 21,018,096.10	–

**Figure 7:**
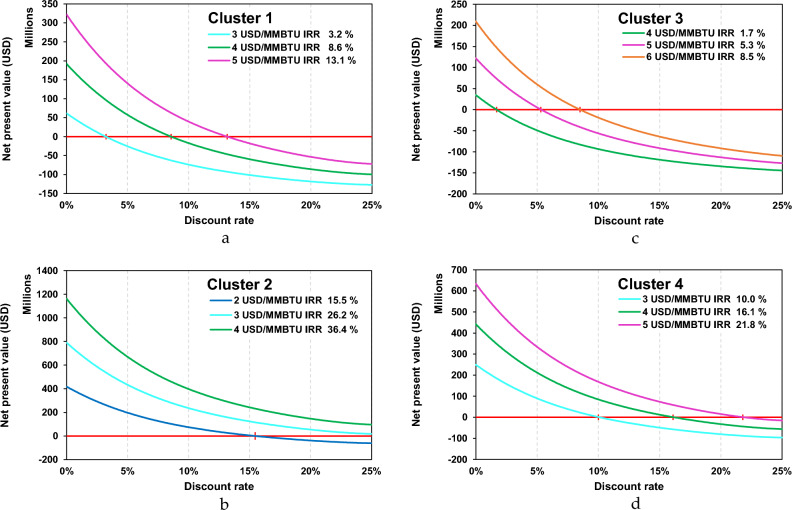
(**a**) NPV and IRR for clusters 1. (**b**) NPV and IRR for clusters 2. (**c**) NPV and IRR for clusters 3. (**d**) NPV and IRR for clusters 4.

**Figure 8:**
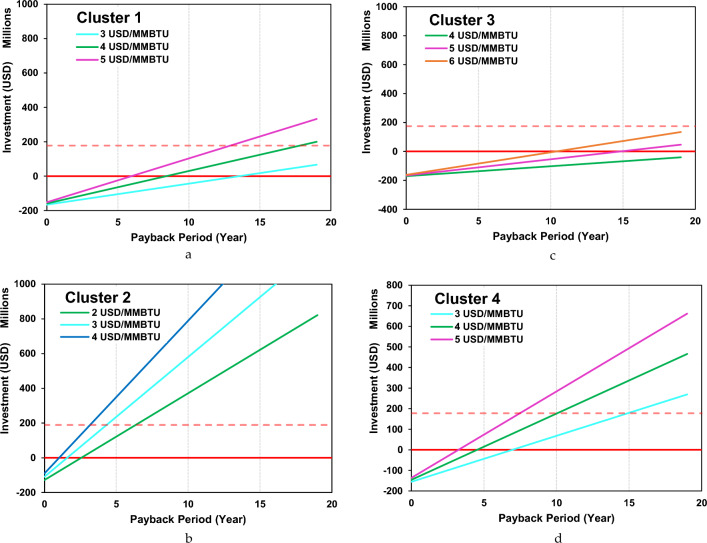
(**a**) Payback period in clusters 1. (**b**) Payback period in clusters 2. (**c**) Payback period in clusters 3. (**d**) Payback period in clusters 4.

The results of NPV and IRR for each cluster are shown in Fig. 7a–d. Thus, the result of the payback period (PP) for each cluster is shown in Fig. 8a–d. The result for cluster 1 shows that the feasible margin rate is 4 USD/MMBTU with IRR 8.6% and the estimated payback period can be achieved within over 15 years. Cluster 2 shows the most interesting results from an economic point of view, in this cluster the feasible margin rate at 2 USD/MMBTU with IRR 15.5% and the estimated payback period can be achieved within before in less than 5 years. On the other hand, in cluster 3, the results are not very attractive from an economic perspective because it has IRR of 8.5% and a fairly long payback period of more than 15 years. In this cluster the feasible margin rate is above 6 USD/MMBTU, this is because the cargo capacity is very small compared to other clusters. Furthermore, cluster 4 shows quite interesting results for development in terms of economics and transportation capacity. In this cluster, the feasible margin rate is 3 USD/MMBTU with an IRR 10.0% and payback period that can be achieved in less than 15 years. From the perspective of small-scale LNG distribution capacity, cluster 4 is the most attractive to develop compared to cluster 1 and cluster 3.

From the results of the economic study, it was found that each cluster has its own characteristics which are influenced by the amount of cargo and the margin level of LNG transportation. The results of economic analysis, it was found that the use of small-scale LNG vessels has a smaller IRR compared to large-scale LNG vessels, on the one hand, the payback period to achieve return on capital cost is longer compared to large vessels. From this economic analysis, it can be concluded that larger the LNG ship is more feasible to invest other than small LNG carriers. With the large LNG ship the margin rate can be pressed and compete with market prices. Based on some literature^61,62^, the value of the margin rate on a large-scale LNG range from 0.65 to 2 USD/MMBTU. However, for LNG distribution with small LNG carriers, several factors exist that cannot be implemented in large LNG distribution transportation activities, e.g. the geographical situation between the terminal facilities, which are far apart and separated by islands. In addition, the sea conditions in eastern Indonesia, which only has a draft below 7 m, are more suitable for small-scale LNG ships from a technical point of view. These results are important to pay attention to, especially for policymakers in determining what incentives should be given so that investors want to run a small-scale LNG distribution business.

### Business strategy and policy initiatives

From the point of view of the economic analysis, as described in section "Principal dimension of Mini-LNG Ships", small-scale LNG distribution is less profitable compared to large LNG distribution since gas prices tend to be lower compared to its transportation cost. It becomes interesting with the case study of the Indonesian region which is an archipelago that has unique challenges in providing electricity with many remote islands that are difficult to reach and have diverse geographical conditions. The government has launched electrification programs aimed at providing electricity access to remote areas and islands to improve the quality of life and support economic development. Regulations and policies are needed that support the supply of electricity in island areas. This includes fair electricity tariff policies, incentives for energy sector investment, and regulations related to renewable energy. In this case study, it is important to know what business strategy and policy initiatives to support LNG distribution for the potential investors.

The majority of the industrial and power generation sectors demand the use of natural gas. Gas supply is high in the renewable energy scenario due to the increase in gas consumption in the power generation sector to meet the surge in household and industrial electrical needs. Therefore, in the current transition period for renewable energy needs, the first policy that can be applied in the developing countries is to develop LNG infrastructure initiatives to accelerate the energy transition process. In addition, the value of LNG emissions is 40% lower than coal. The first initiative that can be taken is to develop the Floating Storage Regasification Unit (FSRU) to maintain the reliability of the natural gas distribution system as a substitution for the existing pipelines on land. When a supply disruption exists, the FSRU can distribute LNG to the receiving station for a gas power plant so that it can maintain the supply of natural gas as needed^63^.

The second policy is to support local governments with the potential for natural gas production by providing LNG infrastructure for power plants in archipelagic areas, such as eastern Indonesia. Increased co-operation between the national companies and the local governments is important in growing a mutually beneficial economy^64^. A form of business co-operation can be done through joint ventures so that the local areas can enjoy their natural resources, build LNG businesses and aid in terms of commercial, technical design and legal aspects.

The third policy is to diversify the use of LNG for transportation fuels. Current developments, with dual fuel diesel and LNG systems, obtain higher efficiency than other fuels. Moreover, the engine efficiency is also higher than other fuels^65,66^. The key factor for LNG as a transportation fuel is the LNG filling station. Infrastructure development can soon realise an LNG filling station terminal at the transportation terminals of sea and land so that commercial LNG can be used by ships’ and commit to reduce emissions.

The fourth policy is LNG for the port area, which has become one of the future businesses. The Indonesian government has issued a regulation related to the implementation of IMO 2020 regarding emission standards with a maximum sulphur content of 0.5%^67^. Most ships still use fuel that produces carbon and sulphur emissions > 0.5%. Thus, an opportunity is taken to provide fuel with lower emissions and 0% sulphur. One of the segments taken is when the ship is in the port area and requires electricity. The technology that can be developed to support this policy is the development of barges as the gas-fired power plants that have an onboard electricity generator with LNG energy sources. The second technology is an electricity shore connection to meet the electricity needs of commercial ships when mooring at the port. By using electricity from the gas-fired power plants, merchant ships will produce zero emissions and can save energy costs of up to 70% at berthing conditions^68^.

The fifth policy is to prepare competitive price regulations in global and domestic markets. One option that can be taken is to reduce the share of the state in the gas or LNG selling price component. Competitive LNG prices can be started with fiscal incentive initiatives, such as exemption from income tax, land and building tax, import duties and indirect taxes or regional levies^69^.

## Conclusion

Techno-economic analysis of the small-scale LNG distribution has been conducted using a capacitated vehicle routing problem. A case study of the gas demands for a gas power plant in eastern Indonesia was optimised into four cluster distribution routes. From the four distribution clusters, optimal ship dimensions are obtained by considering the gas supply capacity for all locations and the aspect ratio from existing small LNG carriers. Each cluster has a different ship capacity that is 5000 cbm, 18,000 cbm, 2500 cbm, and 5000 cbm for cluster 1, cluster 2, cluster 3, and cluster 4, respectively. Of all the ship dimensions has draught are under 10 m which mean can be operated for shallow water depth condition in major remote area and islands.

The results of the economic study show that the feasible margin rate for Cluster 1 is 4 USD/MMBTU with an IRR of 4% and an estimated payback period over 15 years. Cluster 2 with a large gas demand showed that the feasible margin rate is 2 USD/MMBTU with an IRR of 15.5% and an estimated payback period less than 5 years. Cluster 3 with a smaller gas demand showed a feasible margin rate of at least 6 USD/MMBTU with an IRR of 8.5% and an estimated payback period more than 15 years. Cluster 4 has a feasible margin rate of at least 3 USD/MMBTU with an IRR of 10.0% and an estimated PP within 15 years. From the case study of the 4 clusters, it can be concluded that cluster 4 has the potential to develop small-scale LNG distribution where this cluster has a relatively small capacity i.e. 5000 cubic meters, which offers interesting IRR and margin rate. Nevertheless, the economic value remains relatively high in comparison to the current costs of LNG transportation for large volumes of cargo with extremely long interstate trips.

According to the findings of a techno-economic study, the cost of LNG transportation is determined by the number of cargo requests and the shipping distance. Thus, stakeholders must drive small-scale LNG transportation businesses appealing to the investors. The government and shipping companies will be affected by the policy consequences. A minimum transportation cost regulation for each cluster, as well as incentives for clusters with small cargo is needed but high shipping distances, can be enacted by the government. From the standpoint of a shipping company, the policy of using gas as a secondary fuel can save fuel costs. Furthermore, the recommendation for future work is to carry out further techno-economic feasibility of the development of power plants for the island country using various energy sources which are coal, diesel, gas, and renewable energy.

## Data Availability

The author declares: “The datasets used and/or analyzed during the current study available from the corresponding author on reasonable request”.

## Abbreviations

LNG: Liquefied natural gas
CVRP: Capacitated vehicle routing problem
MPP: Mobile power plant
FSRU: Floating storage and regasification unit
MMSCF: Million standard cubic feet
MW: Megawatt
SSLNG: Small-scale LNG carrier
CAPEX: Capital expenditure
OPEX: Operational expenditure
NPV: Net present value
IRR: Internal rate of return
USD: United States dollar
PPI: Producer price index
DWT: Deadweight tonnage
NM: Nautical miles
MMBTU: Million British thermal units
CBM: Cubic metric

## Roman symbols

L: Length over all
B: Breadth/beam
T: Draught/draft
H: Height
Cb: Block coefficient
V: Speeds
